# Changes in Humpback Whale Song Occurrence in Response to an Acoustic Source 200 km Away

**DOI:** 10.1371/journal.pone.0029741

**Published:** 2012-01-11

**Authors:** Denise Risch, Peter J. Corkeron, William T. Ellison, Sofie M. Van Parijs

**Affiliations:** 1 Integrated Statistics, Woods Hole, Massachusetts, United States of America; 2 Northeast Fisheries Science Center, Woods Hole, Massachusetts, United States of America; 3 Marine Acoustics, Inc., Middletown, Rhode Island, United States of America; Institute of Marine Research, Norway

## Abstract

The effect of underwater anthropogenic sound on marine mammals is of increasing concern. Here we show that humpback whale (*Megaptera novaeangliae*) song in the Stellwagen Bank National Marine Sanctuary (SBNMS) was reduced, concurrent with transmissions of an Ocean Acoustic Waveguide Remote Sensing (OAWRS) experiment approximately 200 km away. We detected the OAWRS experiment in SBNMS during an 11 day period in autumn 2006. We compared the occurrence of song for 11 days before, during and after the experiment with song over the same 33 calendar days in two later years. Using a quasi-Poisson generalized linear model (GLM), we demonstrate a significant difference in the number of minutes with detected song between periods and years. The lack of humpback whale song during the OAWRS experiment was the most substantial signal in the data. Our findings demonstrate the greatest published distance over which anthropogenic sound has been shown to affect vocalizing baleen whales, and the first time that active acoustic fisheries technology has been shown to have this effect. The suitability of Ocean Acoustic Waveguide Remote Sensing technology for *in-situ*, long term monitoring of marine ecosystems should be considered, bearing in mind its possible effects on non-target species, in particular protected species.

## Introduction

The last decade has seen an increased awareness of the impacts of anthropogenic underwater noise on marine mammals. Impacts have been described for several different sources, including seismic airguns [1], [2], underwater explosions [3], construction and pile driving [4], acoustic deterrent devices [5], and scientific and military sonar systems [6]–[9]. Possible effects include lethal injuries, short- or long-term hearing damage, and the disruption of normal behavior, including feeding, mating and communication [10]–[11]. Disruption of communication behavior may include signal modifications, for example changes to signal duration, frequency or amplitude [12]–[14], as well as changes in signal usage, repetition, or the cessation of signaling [15], [16], [13], [9]. Changes in communication behavior have been demonstrated across several baleen whale species and in response to various noise sources [17], [2], [14].

This study investigates the effect of low-frequency pulses on the occurrence of humpback whale song. The pulses were produced by an Ocean Acoustic Waveguide Remote Sensing (OAWRS) experiment, roughly 200 km from the whales. The mobile OAWRS system was used to image fish shoals over a 100 km diameter area [18]–[20].

Male humpback whales (*Megaptera novaeangliae*) sing long, complex songs on their breeding grounds [21]. In addition, humpback whales have been shown to sing on migration [22] and feeding grounds [23]. On breeding grounds, humpback whales may alter song production in response to boat noise, seismic surveys and military sonar [24], [8], [25], [26].

Most published examples of the effects of non-chronic anthropogenic noise on marine mammals have dealt with sources within kilometers or perhaps tens of kilometers of the study animals [9]. Effects over hundreds of kilometers have seldom been investigated or demonstrated [27].

Arrays of Marine Autonomous Recording Units (MARUs) [28] gathered low-frequency acoustic data within the Stellwagen Bank National Marine Sanctuary (SBNMS) in 2006 and from December 2007–May 2010 [29], [30]. In autumn 2006, these recordings happened to coincide with an OAWRS experiment in the Gulf of Maine, approximately 200 km distant. Initial perusal of the 2006 data indicated that (a) a novel anthropogenic sound was detected in SBNMS and (b) that humpback whale song in SBNMS occurred less often, coincident with the sound. Despite having before-during-after data for 2006, we could not make inference on the effect of the OAWRS experiment without appropriate control data. Therefore, we collected recordings from approximately the same place, and at the same time, in 2008 and 2009, two years when an OAWRS experiment was not conducted. Thus, despite having what was initially observational data, we configured a design that allowed us to make planned comparisons from our data.

## Materials and Methods

**Figure 1:**
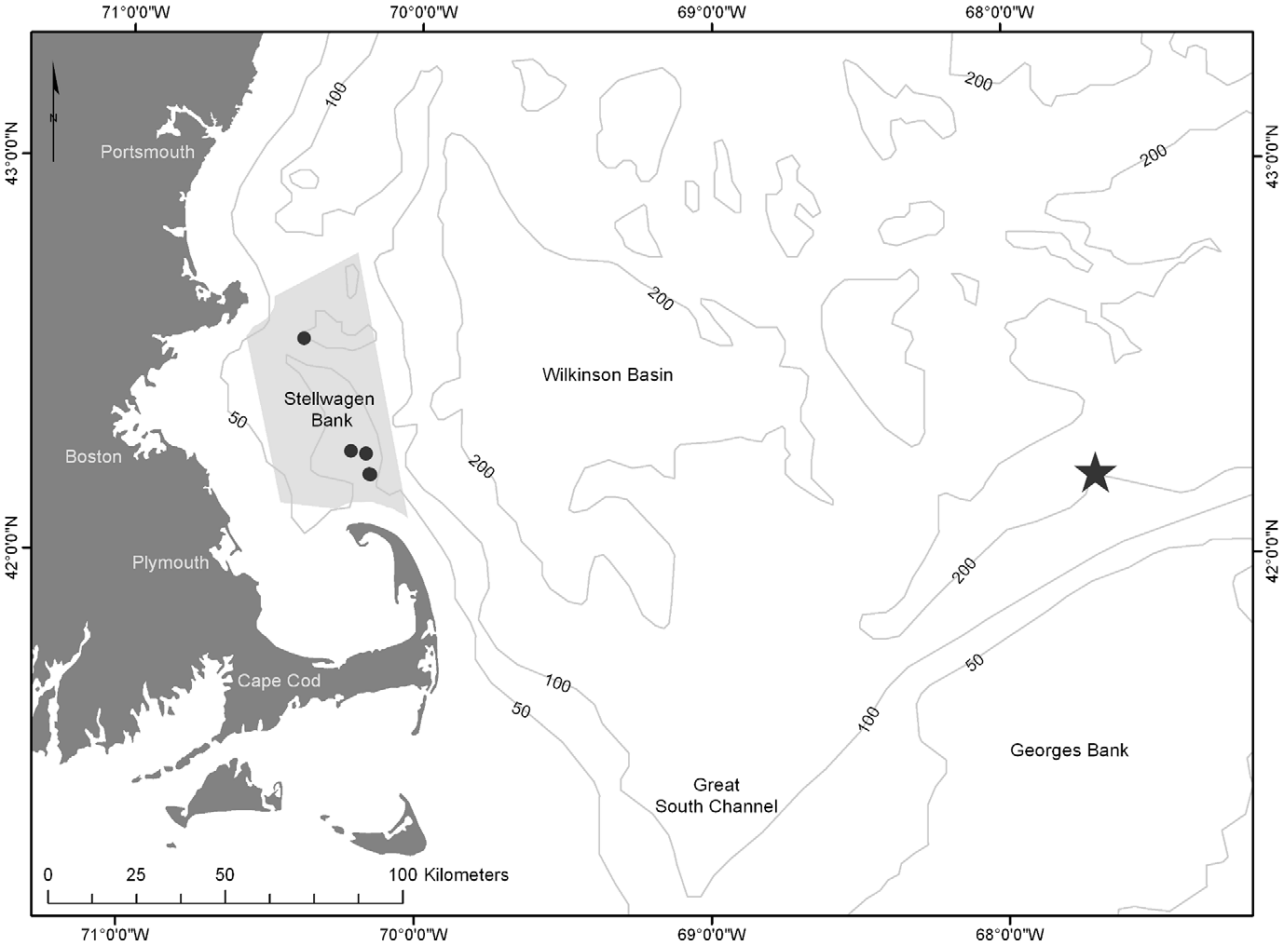
Map of study area (Stellwagen Bank National Marine Sanctuary, shaded in grey) in relation to the location of the moored OAWRS source, as deployed on October 1–3, 2006 (Gong *et al.* 2010). Star indicates approximate OAWRS source location (42.2089 N, 67.6892 W). Dots indicate locations of all MARUs that were used for analysis in 2006, 2008 and 2009. Map projection: Mercator.

Data were collected on arrays of 5–10 MARUs, deployed in SBNMS during September and October of 2006, 2008 and 2009 (Figure 1). Deployments were carried out in cooperation with SBNMS staff and deployment sites were surveyed for archaeological artifacts. Deployment depths ranged from 30–40 m and recorders sampled continuously at a rate of 2000 Hz. Hydrophones were connected to a 23.5 dB preamplifier and had a sensitivity of -168.4 dB re 1 V/µPa. The frequency response was flat (±1 dB) over 55–585 Hz and approximately ±3 dB for 585–1000 Hz.

**Figure 2:**
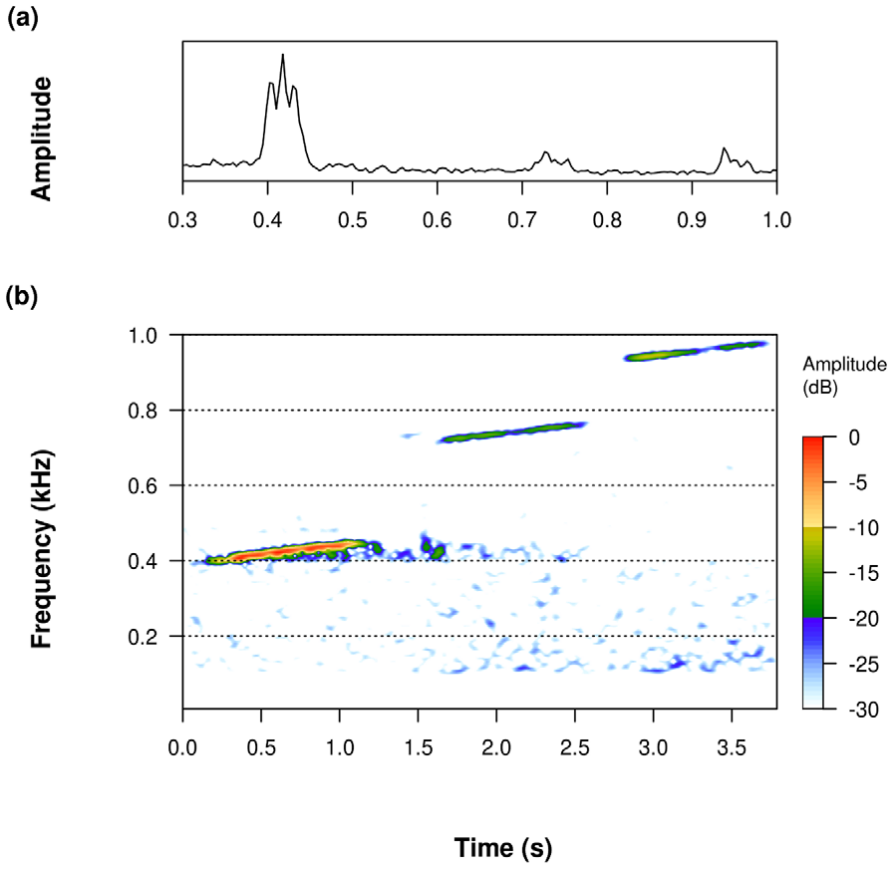
Characteristics of OAWRS signals recorded on MARUs deployed in the Stellwagen Bank National Marine Sanctuary. (a) Mean frequency spectrum, showing local peaks at center frequencies (approx. 415, 735, 950 Hz) of recorded OAWRS FM pulses. (b) Spectrogram (FFT: 512, Hanning window, 75% overlap) of the same pulses as shown in (a). Time interval between successive signals was changed for display purposes; dB scale is relative.

From September 22 to October 6, 2006 we recorded 3 types of frequency modulated (FM) pulses, centered at 415, 734 and 949 Hz, respectively (Figure 2). Based on frequency range and duty cycle, these could be positively identified as FM pulses transmitted as part of an Ocean Acoustic Waveguide Remote Sensing (OAWRS) experiment, conducted in the Gulf of Maine during the same time frame [18]–[20].

For 11 days (September 26 to October 6, 2006) of the 15 day time series, the frequency of occurrence of these pulses exceeded 1 hour/day (Figure S1). We regarded these 11 days as the “OAWRS treatment” period. We determined the number of minutes with humpback whale song/day for a period of 33 days in 2006, encompassing 11 days prior, during and after “OAWRS treatment” (Figure S2). Additionally, presence of song was determined for the same 33 calendar days in 2008 and 2009 (Figures S3, S4).

Spectrograms of sound files were viewed with the software program XBAT [31]. Data from 1 representative MARU were carefully examined (aurally and visually) by an experienced analyst (D Risch). We used 1 MARU, since all simultaneously deployed MARUs spatially overlapped in their detection range for humpback whale song, which in our study area can be detected up to about 30 km [23]. For the purpose of this study, we defined song as consisting of at least 2 full themes, with gaps not exceeding 10 minutes. All instances of song were logged manually. An automated template detector in XBAT was used to find instances with OAWRS FM pulses in the 2006 data and characterize their temporal occurrence. The detector assessed acoustic similarity between a data template and possible events by spectrogram cross-correlation and logged all events exceeding a correlation threshold of 0.4. Automatically detected events were manually checked to verify signal presence and signals that were missed by the detector were logged manually.

Spectral, temporal and received level (RL) measurements of OAWRS pulses were made in Seewave [32] and Raven Pro 1.4 (http://www.birds.cornell.edu/raven, accessed 7 June 2011) using a Fast Fourier Transform (FFT) length of 512 samples, 75% overlap and Hanning window, giving a time and frequency resolution of 64 ms and 4 Hz, respectively. OAWRS signal RLs (dB re 1 µPa) were calculated by measuring dB RMS over an event box (approx. 380–440, 710–760, 930–980 Hz; 1 s). Using the same time and frequency bounds, background noise levels (NL) were measured 50 ms before or after each event for windows without the signal. Subsequently, signal-to-noise ratio (SNR) was calculated by subtracting NLs from signal RLs.

To assess changes in background noise other than the occurrence of OAWRS pulses in 2006, and as compared to the two control years, ambient sound levels in frequency bands covering the frequency range of our recording system (10–1000 Hz) as well as in the frequency band with most humpback whale song energy (70–300 Hz, pers. obs.) were measured over the entire analysis period using a customized Matlab script (LTSpec, K. Cortopassi, unpublished).

Statistical analysis was conducted using R 2.13.2 [33]. We used a quasi-Poisson generalized linear model (GLM) with log link to test the effects of period (11 days: ‘before’, ‘during’, ‘after’) and year (‘2006’, ‘2008’, ‘2009’) on the number of minutes with humpback whale song. The OAWRS pulses were recorded only during 2006. The other years serve as controls in the temporal equivalent of a BACI design [34]. This was a planned comparison, as we noted a possible effect in 2006, and collected control data in 2008 and 2009 in response to this possibility.

GLMs assume the independence of response variables. Since we analyzed a time series of singing behavior of possibly the same individuals, we checked for residual correlation and plotted temporal autocorrelation of our data. No temporal correlation of residuals was found (Figure S5). Tukey contrasts were calculated from the fitted model to test for differences between periods across and within years, using the function ‘glht’ in R package ‘multcomp’ [35].

## Results

The FM pulses recorded in SBNMS from September 26 to October 6, 2006 had a bandwidth of roughly 50 Hz, duration of 1 s, and mean center frequencies of 415, 734 and 949 Hz (Figure 2, Table 1). FM pulses of each center frequency were recorded every 150 s. FM pulses centered at 415 and 734 Hz were recorded seconds apart, followed by the pulse centered at 949 Hz after 75 s. The frequency range and duty cycle of these pulses allowed their positive identification as pulses produced during the OAWRS 2006 experiment in the Gulf of Maine [18]–[20]. A fourth pulse centered at 1125 Hz was transmitted during this experiment but was not recorded by our system, which was limited to an effective recording bandwidth of 1000 Hz.

**Table 1:** Summary of OAWRS FM pulse characteristics (mean±SD), as measured from spectrograms (FFT: 512 samples, Hanning window, 75% overlap) and waveforms of MARU recordings on October 1–3, 2006 (sample rate: 2000 Hz, recording depth: 30–40 m).

	FM 1	FM 2	FM 3
Signal duration (s)	1.0±0.1	1.0±0.1	1.0±0.1
Low Frequency (Hz)	388.3±2.0	709.1±2.7	923.5±2.8
High Frequency (Hz)	441.2±2.2	759.3±3.7	972.4±3.6
Bandwidth (Hz)	52.8±2.7	50.2±3.7	50.5±3.5
Center Frequency (Hz)	414.8±7.0	733.6±7.0	948.7±6.3

N = 60.

A total of 83 hours of recordings contained OAWRS pulses (mean ± SD: 8±3 hours/day, n = 11 days), with more than 7 hours of signal occurrence/day from September 27 to October 4, 2006 (see Figure S1). The OAWRS source array was deployed at the northern flank of Georges Bank (42.2089 N, 67.6892 W), about 200 km from our bottom-mounted acoustic recorders at the western edge of Stellwagen Bank (Figure 1) [19]. Signal RLs on these days ranged from 88–110 dB re 1 µPa (Table 2). Over the 99 days for which data were collected, there were 219.9 hours of humpback whale song recorded.

**Table 2:** Received level (RL) measurements over full bandwidth of OAWRS FM pulses, ambient noise (NL) measurements over the same bandwidths, signal-to-noise ratios (SNR) and signal excess (SE) (mean±SD).

	FM 1	FM 2	FM 3
Center Frequency (Hz)	415	734	949
RL Signal (dB re 1 µPa)	110.3±3.3	88.0±3.2	89.8±3.3
NL Ambient (dB re 1 µPa)	88.0±3.3	82.9±2.6	81.6±2.5
SNR = RL-NL (dB)	22.3±4.8	5.1±4.0	8.2±3.9
SE = SNR-10 dB	12.3±4.8	−4.9±4.0	−1.8±3.9

N = 677.

**Figure 3:**
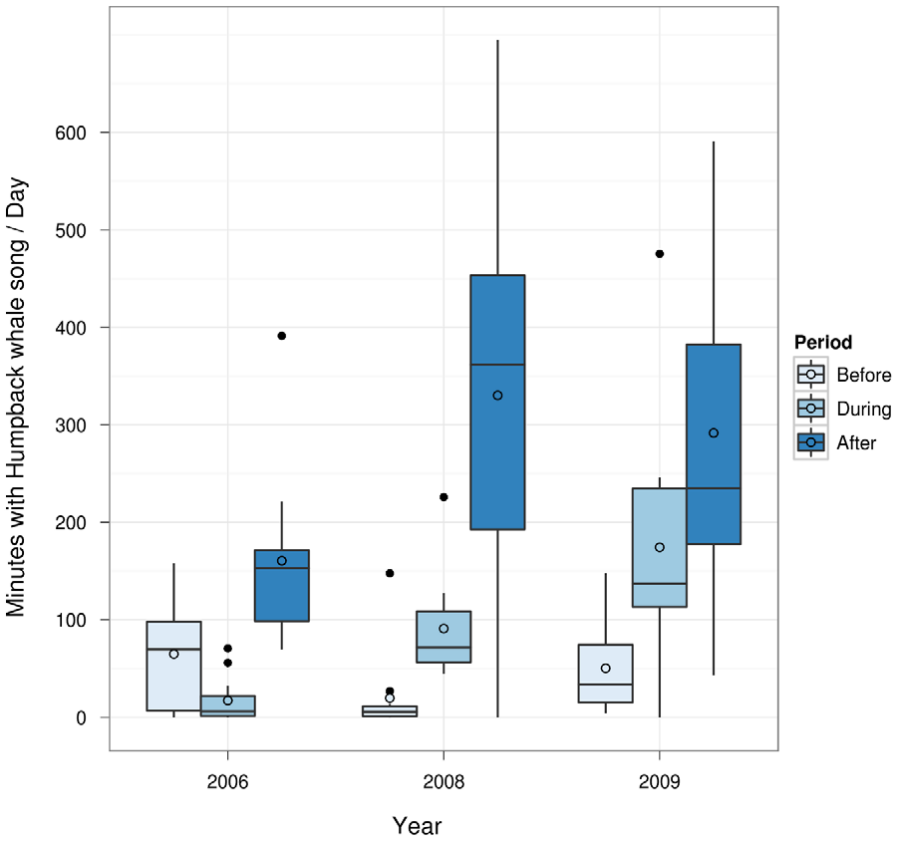
Box-and-Whisker plot of minutes/day containing humpback whale song for 33 days ‘before-during-after’ OAWRS FM pulse transmissions in 2006, and for the same 33 calendar days in 2008 and 2009. Lower and upper bounds of boxes represent lower and upper quartiles, respectively. Solid lines represent medians and non-filled circles are means. Whiskers represent furthest data points within 1.5× interquartile range (IQR) of the lower and higher quartile, respectively. Filled dots are outliers.

The amount of recorded humpback whale song differed between periods and years. The occurrence of song in the control years increased steadily across the three test periods; conversely there was a marked decrease in the occurrence of song in 2006 in the ‘during’ period, when the OAWRS transmission was recorded, that was not evident in the control years (Figure 3). While the ‘before’ and ‘after’ periods differed significantly within the years 2008 and 2009 (Figure 3, Tukey contrasts, P<0.001), with more song recorded in the later period in both years, this increase was not significant in 2006 (P = 0.2147). In 2006, the ‘during’ period, (i.e. during the OARWS experiment), was significantly different from the period ‘after’ (P = 0.0093), with more song recorded later. The 2006 ‘during period’ was not detectably different from the period ‘before’ (P = 0.5226). When comparing the ‘during’ period across years, 2006 differed significantly from 2009 (P = 0.0057). The same time period did not differ significantly between 2006 and 2008 (P = 0.1842), or between 2008 and 2009 (P = 0.4819). Yet, overall there was considerably less song recorded in the 11 ‘during’ days in 2006 compared to both 2008 and 2009 (Figure 3). Throughout the whole analysis period, ambient noise levels in the 70–300 Hz and 10–1000 Hz frequency band were within 4 dB of each other [mean(70–300 Hz) ± SD: 107.7±3.8 dB re 1 µPa; mean(10–1000 Hz) ± SD: 114.6±3.5 dB re 1 µPa; n = 99 days].

## Discussion

In general, we detected humpback whale song less in our study area concurrent with OAWRS signal transmissions than at other times. The RLs of OAWRS pulses approximately 200 km from the source array were 5–22 dB above ambient noise levels. Pulses centered at 415 Hz had a mean SNR of 22.3 dB. For pulses at 734 Hz and 949 Hz mean SNR was 5.1 and 8.2 dB, respectively (Table 2). Signal detection in background noise is usually not at SNR = 0 dB, but is dependent on a receiver characteristic, the detection threshold (DT). The difference between SNR and DT is signal excess (SE). A nominal DT value of 10 dB is well supported in the current literature [30]. In common practice, the value of SE = 0 is established at the point of 50% detection probability. In application to our data, SE for pulses at 415, 734 and 949 Hz was 12.3, −4.9 and −1.8 dB, respectively (Table 2). With SE values slightly lower than 0 dB the detection of the two FM pulses with higher center frequencies was probably right on the edge of perception for humpback whales in our study area. For the pulse at 415 Hz SE was still relatively low at 12 dB.

Thus, in response to OAWRS FM pulses, with relatively low SE, male humpback whales either moved out of the study area or sang less. Our data were collected using passive acoustic monitoring, so we cannot differentiate between these two options. However, although very limited, visual data collected in SBNMS before, during and after the 2006 experiment give more weight to the second alternative. Several known, sexually mature males (ages 6–28 years) were photographically identified in SBNMS during the OAWRS experiment. While only two known males were identified prior to the experiment, four individuals were present in the area in the “during” period (J. Robbins, pers. comm.). This suggests that individuals did not leave the area but instead ceased singing. Multi-year data from SBNMS [36] show that humpback whale song generally increases at the end of summer and into early winter, when the whales start to migrate south.

Ambient noise levels over the whole analysis bandwidth (10–1000 Hz) and in the frequency band with most humpback whale song energy (70–300 Hz) did not vary dramatically within or between years. However, the drop in humpback whale song, recorded during the OAWRS experiment in October 2006, was not repeated in the two control years (Figure 3). Therefore, our data provide clear evidence for the reduction of humpback whale song in response to the reception of OAWRS pulses. We interpret this decrease as a change in singing behavior by individual whales.

Several large whale species have been shown to stop vocalizing in response to anthropogenic noise. For example, sperm (*Physeter macrocephalus*) and blue whales (*Balaenoptera musculus*) reacted to seismic survey activities with silence [15], [37]. Blainville's beaked whales have recently been shown to avoid ships using active mid-frequency sonar and decrease the duration of vocal periods during sonar exercises [9].

Current approaches to management of anthropogenic noise in marine mammal habitats are predicated on a dose-response model, based on maximum RLs proximate to the source [11]. However, the alteration of male humpback whale song in SBNMS in response to sounds with low SE values, received roughly 200 km from the source, suggests that factors other than absolute RLs must also be considered when assessing the effects of anthropogenic sound on marine mammals. Behavioral change in response to low levels of noise is likely strongly dependent on the behavioral state of the individual as well as the exposure context (i.e. proximity, encroachment, novelty, including similarity to other biologically relevant signals) [38]. Given the short duration of the OAWRS experiment, the novelty of the FM pulses to humpback whales in SBNMS in particular provides a compelling contextual probability for the observed effects. In addition, OAWRS pulses overlap with humpback whale sounds in frequency band (400–900 Hz), duration (1 second) and signal type (FM). This acoustic similarity paired with a relatively low signal excess (SE) might have been another factor driving the observed behavioral or distributional changes. These findings stress the importance of adding contextual information to behavioral assessments of noise impacts. They also illustrate the requirement to both measure and assess background noise [38].

We initially detected this behavioral effect serendipitously. However, our ability to make inference on its existence is thanks to our (within year) before-during-after and (between year) control-impact design. To our knowledge, no-one has tested for behavioral effects of sound on whales at distances of greater than tens of kilometers. Our results suggest that this is an oversight.

In the absence of effective far field source level (SL) data, we cannot make inference on the effects of the OAWRS signal on those humpback whales that may have been closer to the sound source than our study site. Yet, Gong *et al.* [39] recorded marine mammal vocalizations, presumably humpback whales, on George's Bank much closer to the source (Figure 1), concurrent with the 2006 OAWRS experiment. However, as these authors present no data on humpback whales' use of George's Bank at any time other than during this experiment it is difficult to make inference on its effect on humpback whale behavior at these closer spatial ranges. The response of individuals can also be variable. In a playback experiment using low-frequency active (LFA) sonar, Miller *et al.* [8] showed that, on average, humpback whale songs were longer during playback as compared to before or after control periods. Yet, these authors also noted the cessation of singing by 5 of their 18 focal animals in response to the playback. Due to differences in behavioral context, location and proximity to the sound source it is difficult to directly compare our findings to either of the mentioned studies. However, it is worth noting that plasticity in behavioral responses is likely to exist on several different levels, including the individual level.

The current paradigm for assessing effects of anthropogenic noise is for short-term, short distance experiments, with a focus on acute events and the absolute level of received sound. Our results indicate that longer-term, larger scale monitoring of anthropogenic sound is also necessary.

## Supporting Information

Figure S1 Time series of hourly detections of OAWRS signals recorded on MARUs deployed in the Stellwagen Bank National Marine Sanctuary in September/October 2006.
(TIF)

Figure S2 Time series of minutes with humpback whale song detections in September/October 2006. Plot is split in three panels representing (a) ‘Before’, (b) ‘During’ and (c) ‘After’ periods. Right y-axis displays date.
(TIF)

Figure S3 Time series of minutes with humpback whale song detections in September/October 2008. Plot is split in three panels representing time periods equal to (a) ‘Before’, (b) ‘During’ and (c) ‘After’ periods in 2006. Right y-axis displays date.
(TIF)

Figure S4 Time series of minutes with humpback whale song detections in September/October 2009. Plot is split in three panels representing time periods equal to (a) ‘Before’, (b) ‘During’ and (c) ‘After’ periods in 2006. Right y-axis displays date.
(TIF)

Figure S5 (a) Plot of residuals of quasi-poisson GLM model for OAWRS data. (b) Temporal autocorrelation plot based on residuals of quasi-poisson GLM model used in OAWRS analysis. Blue dashed line indicates approximate 95% confidence interval.
(TIF)
