# Supplementary figures and images for: Changes in Humpback Whale Song Occurrence in Response to an Acoustic Source 200 km Away
Source: PLoS One. 2012 Jan 11;7(1):e29741. doi: 10.1371/journal.pone.0029741 (PMC3256173; doi:10.1371/journal.pone.0029741)

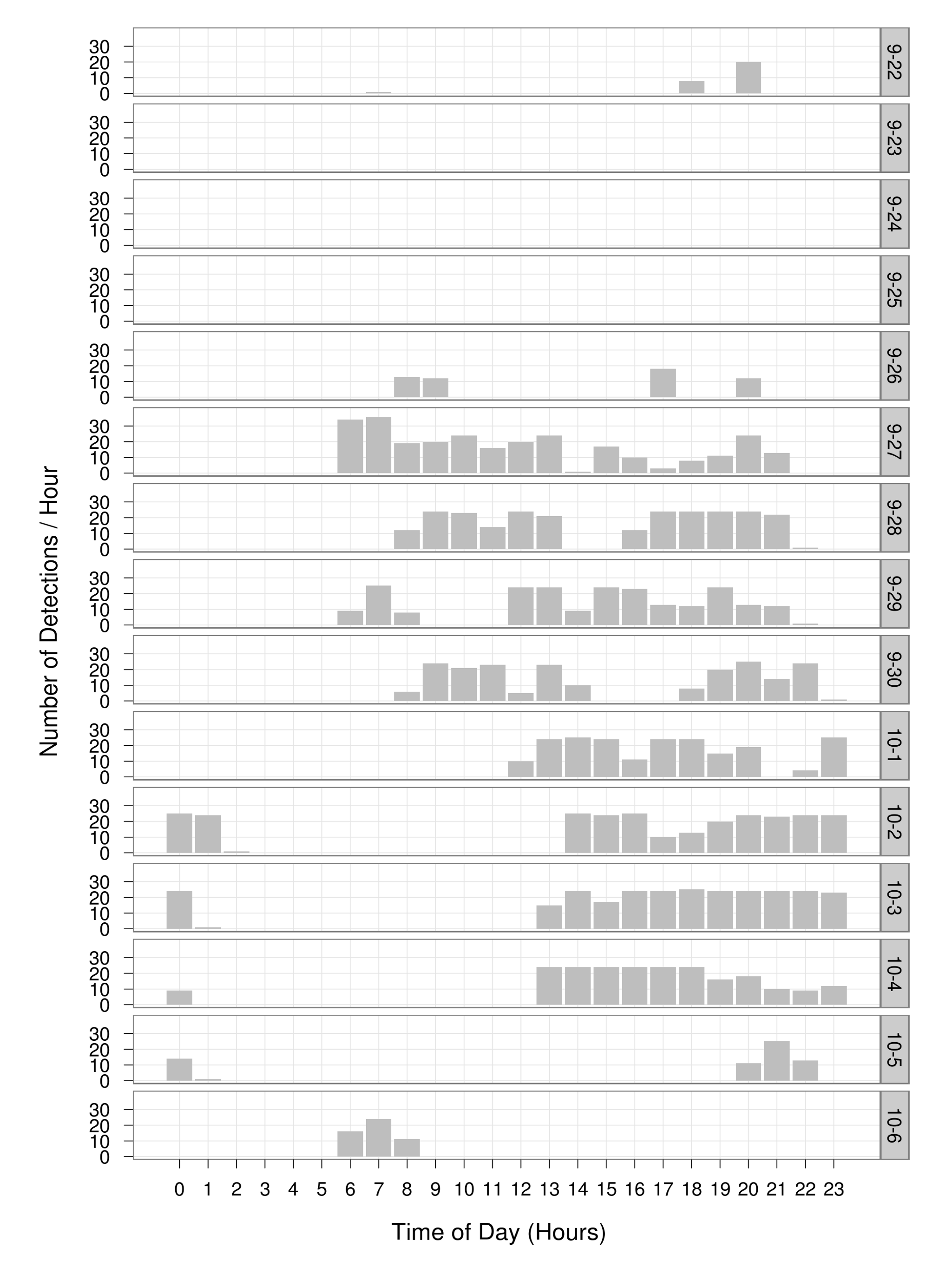

Supplement: Figure S1 — Time series of hourly detections of OAWRS signals recorded on MARUs deployed in the Stellwagen Bank National Marine Sanctuary in September/October 2006. (TIF) [file pone.0029741.s001.tif]

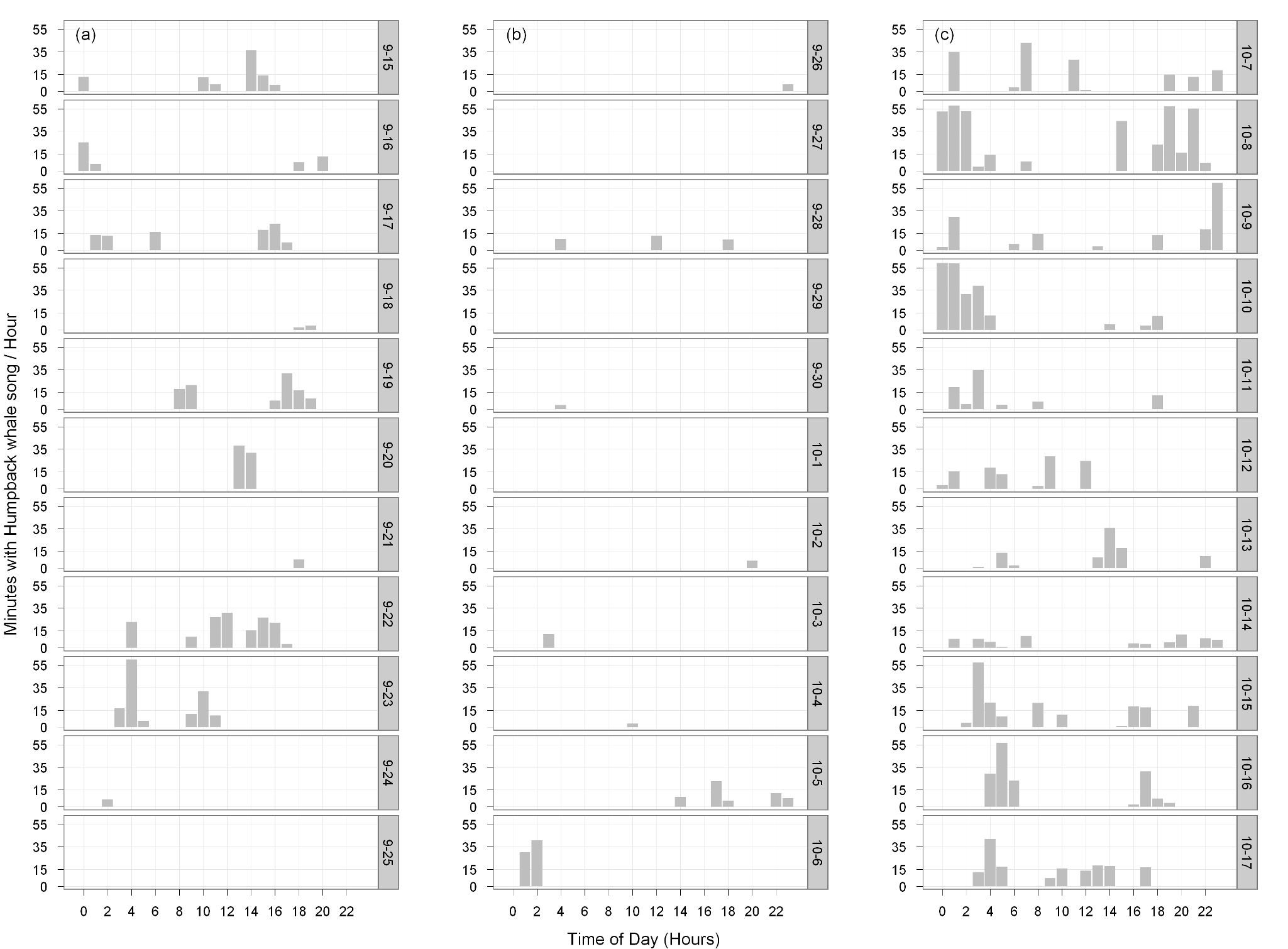

Supplement: Figure S2 — Time series of minutes with humpback whale song detections in September/October 2006. Plot is split in three panels representing (a) ‘Before’, (b) ‘During’ and (c) ‘After’ periods. Right y-axis displays date. (TIF) [file pone.0029741.s002.tif]

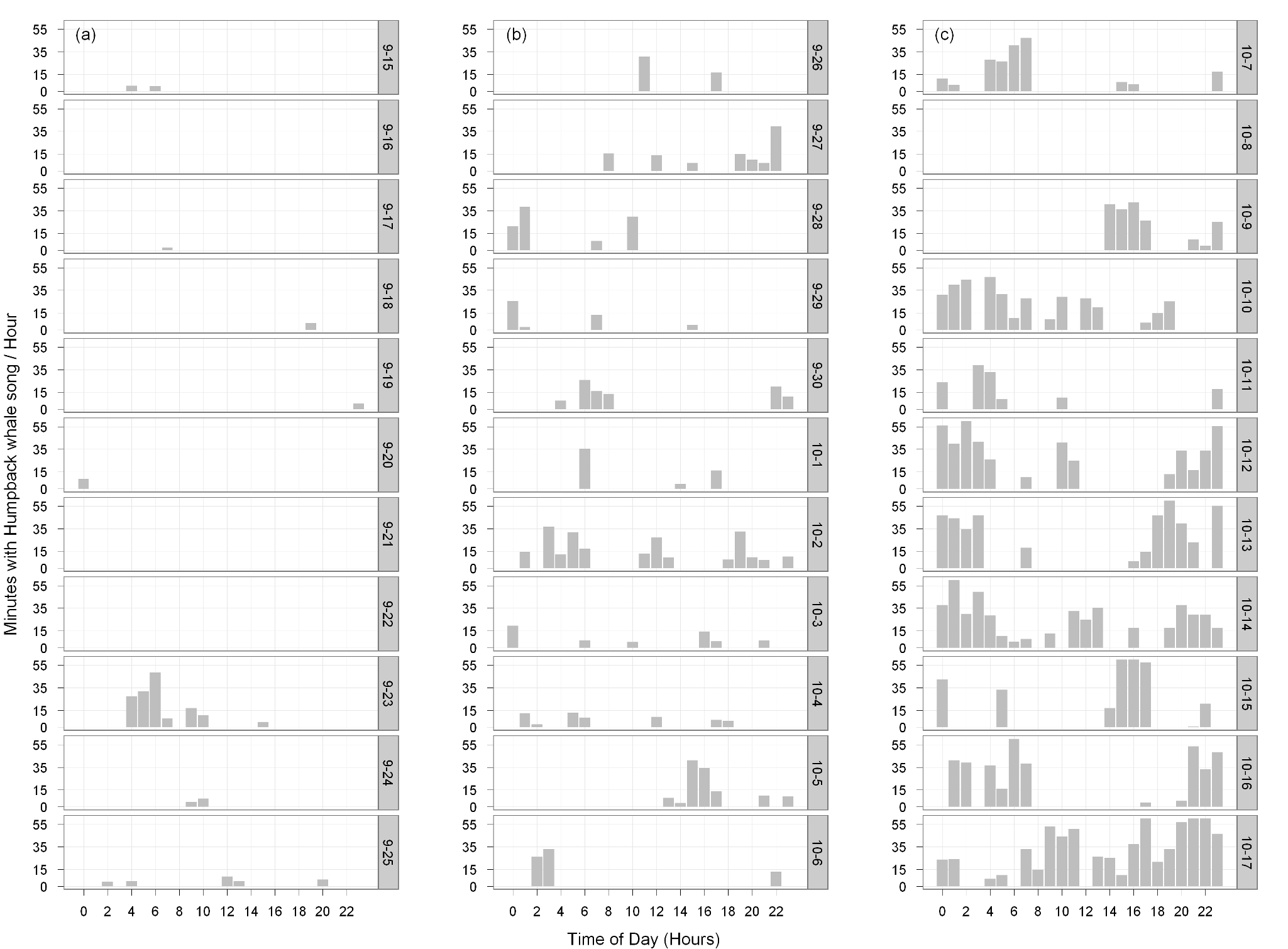

Supplement: Figure S3 — Time series of minutes with humpback whale song detections in September/October 2008. Plot is split in three panels representing time periods equal to (a) ‘Before’, (b) ‘During’ and (c) ‘After’ periods in 2006. Right y-axis displays date. (TIF) [file pone.0029741.s003.tif]

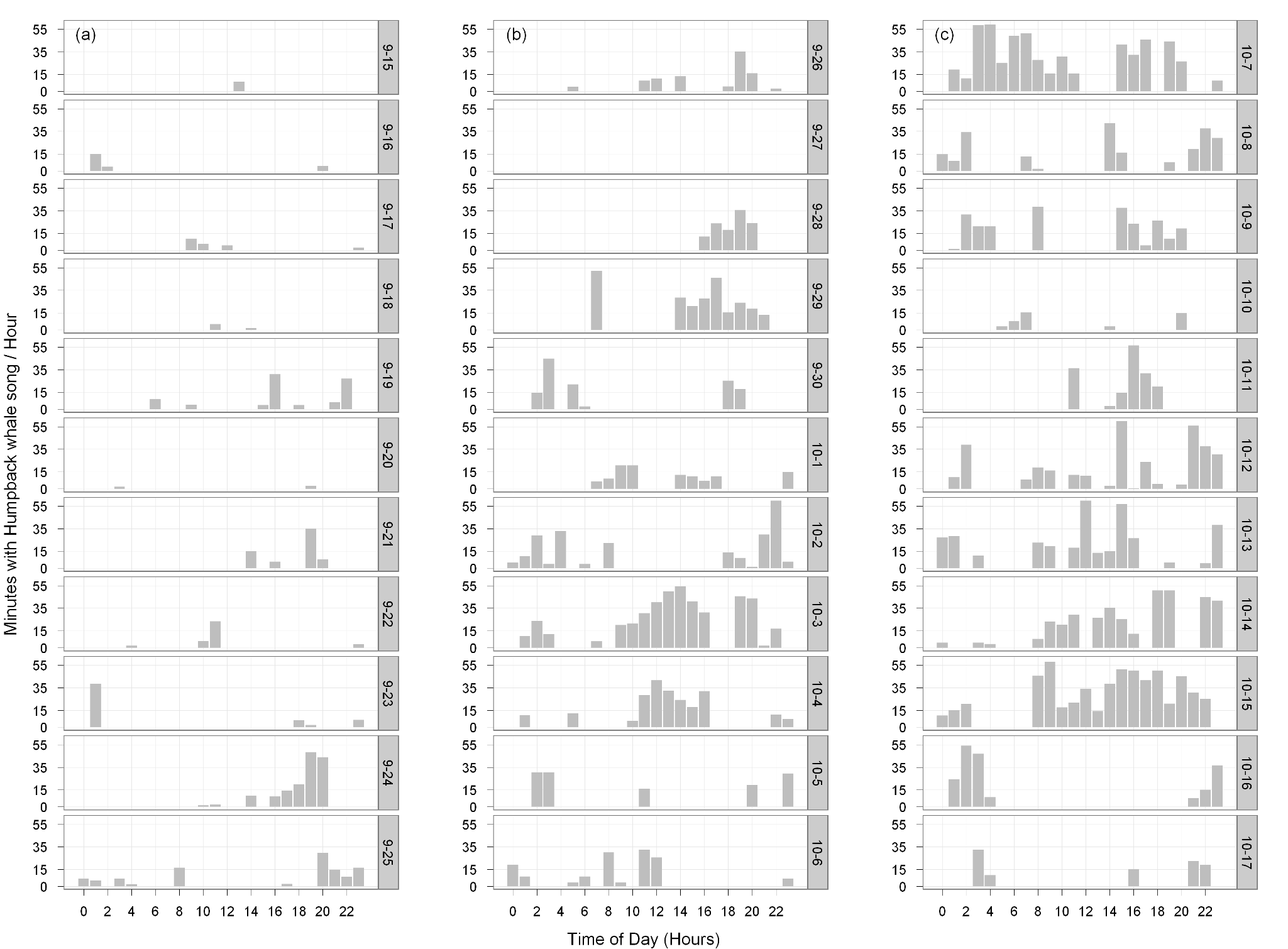

Supplement: Figure S4 — Time series of minutes with humpback whale song detections in September/October 2009. Plot is split in three panels representing time periods equal to (a) ‘Before’, (b) ‘During’ and (c) ‘After’ periods in 2006. Right y-axis displays date. (TIF) [file pone.0029741.s004.tif]

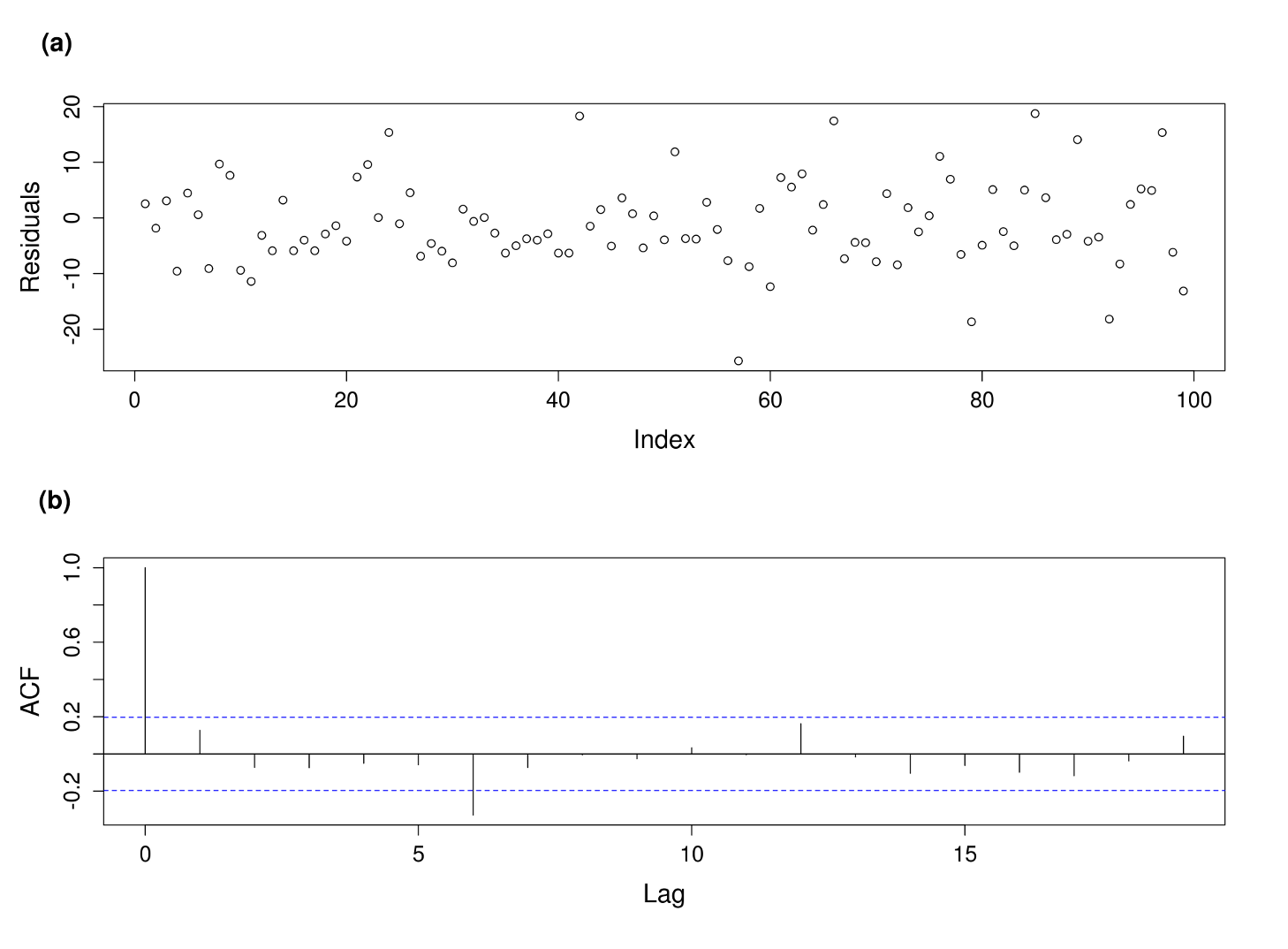

Supplement: Figure S5 — (a) Plot of residuals of quasi-poisson GLM model for OAWRS data. (b) Temporal autocorrelation plot based on residuals of quasi-poisson GLM model used in OAWRS analysis. Blue dashed line indicates approximate 95% confidence interval. (TIF) [file pone.0029741.s005.tif]
